# Association of Serum Albumin and Copeptin with Early Clinical Deterioration and Instability in Community-Acquired Pneumonia

**DOI:** 10.3390/arm90040042

**Published:** 2022-08-10

**Authors:** Ashwaghosha Parthasarathi, Vaibhav C. Padashetti, Sunag Padukudru, Sindaghatta Krishnarao Chaya, Jayaraj Biligere Siddaiah, Mahesh Padukudru Anand

**Affiliations:** 1 Allergy, Asthma, and Chest Centre, Krishnamurthypuram, Mysore 570004, India; 2 Department of Respiratory Medicine, JSS Medical College, JSSAHER, Mysore 570015, India; 3 Yenepoya Medical College, Yenepoya University, Mangalore 575018, India

**Keywords:** copeptin, albumin, risk stratification, community-acquired pneumonia, biomarkers

## Abstract

**Highlights:**

**What are the main findings?**

- Serum albumin lower than 2.85 g/dL is the best predictor of mortality in CAP patients
- The addition of serum albumin and copeptin to the traditional clinical scoring systems increases it’s prognostic power

**What is the implication of the main finding?**

- Biomarkers serum albumin and copeptin can predict early deterioration and clinical instability in hospitalized CAP patients
- Serum albumin, copeptin and ATS/IDSA criteria should be used to predict mortality in patients for predicting clinical instability after 72 h, ICU admission and mortality at day 7 after hospital admission

**Abstract:**

Background: There is a paucity of data on biomarkers for the early deterioration and clinical instability of patients in community-acquired pneumonia (CAP), as treatment failure occurs in the first seven days in 90% of patients. Aim: To evaluate serum albumin and copeptin with CURB-65, PSI scoring and ATS/IDSA minor criteria for the prediction of early mortality or ICU-admission (7 days) and clinical instability after 72 h. Methods: In 100 consecutive hospitalized adult CAP patients, PSI-scores, CURB-65 scores, ATS/IDSA 2007 minor criteria, copeptin and albumin on admission were evaluated. Univariate and multivariate Cox regression analysis was performed to assess independent risk factors for early combined mortality or ICU admission. Predictive powers of albumin and copeptin were tested with ROC curves and ICU-free survival probability was tested using Kaplan–Meier analysis. Results: Albumin was lower and copeptin higher in patients with short-term adverse outcomes (*p* < 0.05). Cox regression analysis showed that albumin [HR (95% CI): 0.41 (0.18–0.94, *p* = 0.034)] and copeptin [HR (95% CI): 1.94 (1.03–3.67, *p* = 0.042)] were independent risk factors for early combined mortality or ICU admission (7 days). The Kaplan–Meier analysis observed that high copeptin (>27.12 ng/mL) and low albumin levels (<2.85 g/dL) had a lower (*p* < 0.001) survival probability. The diagnostic accuracy of albumin was better than copeptin. The inclusion of albumin and copeptin into ATS/IDSA minor criteria significantly improved their predictive power. Conclusions: Both biomarkers serum albumin and copeptin can predict early deterioration and clinical instability in hospitalized CAP patients and increase the prognostic power of the traditional clinical scoring systems.

## 1. Clinical Rationale for the Study

Community-acquired pneumonia is an important cause of death globally and there is a need to identify biomarkers for triaging and identifying patients at risk for clinical instability and mortality. Currently, clinical scores such as the pneumonia severity index (PSI) and the ATS/IDSA criteria are used, but previous studies have demonstrated that there is scope for improvement. There is a need for additional biomarkers that could improve upon the accuracy of the current scoring systems. We therefore evaluated one commonly evaluated biomarker, which is routinely measured in almost all admitted patients with community-acquired pneumonia and serum copeptin, which is not routinely used in the clinical setting to understand whether these biomarkers confer additional accuracy when added to routinely used clinical tools such as the PSI and ATS/IDSA scores.

## 2. Introduction

Community-acquired pneumonia (CAP) is a relatively common respiratory infection with high morbidity and mortality [1] despite the development of novel interventions [2]. Globally, although age-standardized death rates fell by 19.5 percent, mortality due to LRTI remained unchanged from 2005 to 2015 [3]. There has been a steady increasing trend in hospitalization rates, including intensive care units (ICU), due to CAP, especially in the elderly [4]. The case fatality rate for CAP has been known to reach 50 percent in patients admitted to ICUs, with a range of 2% to 20%. It differs according to age, geography, comorbidities and healthcare settings [5]. Alarmingly, India shoulders almost one-fourth of the global pneumonia burden, with approximately four million cases of CAP per year [6,7].

International guidelines recommend the use of prognostic tools such as the pneumonia severity index (PSI) and CURB-65 (confusion, urea, respiratory rate, blood pressure and age ≥ 65) scores to support clinical decisions [8,9]. These established scoring systems have been proven to be effective in the identification of low-risk patients who can be managed in an outpatient setting. They also perform adequately in predicting CAP-associated mortality [10,11].

A patient with CAP presenting with respiratory failure requiring mechanical ventilation or septic shock with need for vasopressors, ICU admission and intensive management is easy to recognize [12]. Nonetheless, if a patient does not present with these major criteria, it becomes a challenge to predict clinical instability and deterioration. The traditional scoring systems, i.e., CURB-65 and PSI, perform poorly in this regard [13]. Thus, additional tools, such as the American Thoracic Society (ATS)/Infectious Diseases Society of America (IDSA) major and minor criteria, are being used to identify early intensive management [12]. These criteria, however, have been found to miss a significant portion of patients needing intensive management and ICU admission [13,14].

Biomarkers are biological markers that can be measured accurately and reliably reproduced. It has been used extensively for various disease conditions [15,16]. The most commonly used biomarkers in CAP are C-reactive protein (CRP) and procalcitonin (PCT). Others with less evidence include pro-atrial natriuretic peptide (pro-ANP), pro-vasopressin (pro-VNP), soluble triggering receptor expressed on myeloid cells (sTEM), pro-adrenomedullin (pro-ADM), surfactant protein-D (SP-D) and endocan barrier-stabilizing angiopoietins 1 and 2 (Ang 1 and Ang 2) [17].

In CAP, numerous studies have assessed the role of biomarkers for different outcomes. These include the diagnosis of pneumonia, mortality risk stratification, severity of illness and initiation and duration of antibiotic therapy [18]. However, there is a paucity of data on early mortality/ICU admission and clinical instability. This is extremely important as research has shown that the early identification of severe CAP significantly improves treatment outcomes [19].

It was demonstrated that treatment failure related to CAP occurs within the first 7 days in more than 90% of cases, while 9 out of 10 patients who have succumbed to CAP-related failure do so in the first seven days of admission [20]. Thus, the aim of our study was to evaluate the use of serum albumin and copeptin in comparison and combination with CURB-65, PSI scoring and ATS/IDSA minor criteria for the prediction of an early (7 days) mortality/ICU admission and clinical instability within 72 h of admission in hospitalized CAP patients.

## 3. Methodology

### 3.1. Study Population

We conducted an observational study in a tertiary care hospital from March 2019 to January 2020. This study was approved by the Institutional Ethics Committee of JSS Medical College, Mysuru (Approval number: JSS/MC/PG/4623/2018-19). A total of 100 consecutive adult CAP patients were enrolled in the study.

CAP was defined by the criteria laid out by Niederman et al. [21]:

- Chest X-ray with patchy infiltrates, segment/leaf consolidation, interstitial change or ground-glass opacity;
- At least one of the following signs:
  ◦ Symptoms of cough, phlegm, or dyspnea;
  ◦ Temperature greater than 38.0 °C;
  ◦ Abnormal auscultatory findings such as abnormal breath sounds and rales;
  ◦ Leukocytosis (greater than 10 × 10^9^/L).

- The onset of symptoms should be in a community setting, not a health care setting.

Patients with chronic kidney disease, liver disease, pregnancy, lactation, malignancy, active infection other than pneumonia, sepsis not secondary to CAP, i.e., other infection, renal failure, burns, malabsorption syndromes, malnutrition status and rheumatic disease, cardiovascular diseases acute or chronic, bronchial carcinoma, diabetes insipidus (DI) and those who failed to give written informed consent were excluded from the study.

### 3.2. Laboratory and Clinical Assessments

Demographic data from patient age, gender, length of hospital stay, body temperature and clinical features were recorded. Hematological panel performed on an automated blood analyzer “Sysmex XN 1000” was recorded. Absolute neutrophile count/absolute lymphocyte count (NLR) and platelet count/absolute lymphocyte count (PLR) were calculated. Pneumonia severity index (PSI), CURB-65 and the minor criteria for ICU admission by ATS/IDSA guideline were assessed [8,9,20].

On day of admission, 5 mL of venous blood was obtained in an anticoagulant-free plain tube and was allowed to clot at room temperature for 30 min. This was centrifuged at 1000 rpm for 15 min, and serum samples were stored at −80 °C. Serum copeptin and albumin collected within 24 h of admission were assessed using ELISA kits (KinesisDx, Brea, CA, USA). Antibiotic administration and the treatment protocol was adopted from the ICMR guidelines for treatment of community-acquired pneumonia [22].

The outcomes assessed were clinical instability within 72 h after admission and ICU admission or mortality at day 7 of hospital admission. Clinical instability was defined as the presence of at least one of the following criteria as laid out by Halm et al. [23], i.e., heart rate greater or equal to 100/min, respiratory rate greater or equal 24/min, systolic blood pressure lesser than or equal 90 mmHg, temperature greater or equal to 37.8 °C, inability to eat, oxygen saturation lesser than or equal 90% and inability to return to baseline mental status.

### 3.3. Statistical Analysis

Statistical analysis was performed employing JAMOVI (v1.6, The jamovi project, Sydney, Austrilia). Continuous variables were presented as either mean ± standard deviation if they were normally distributed or median with their interquartile range if not normally distributed. Categorical variables were presented as percentages. Statistical significance was assessed by chi-square test for categorical variables and by Student’s *t* or Wilcoxon test for continuous variables depending on the distribution of data. Similarly, Pearson’s r test for correlation was used to assess normally distributed data whereas non-normally distributed data were assessed using Spearman’s rho test.

Cox proportional hazards regression analyses and Kaplan–Meier method was used to draw up 7-day survival curves, while the survival rates were compared using the log-rank test. The area under the curve (AUC), sensitivity, specificity, odds ratio and optimal cut-off values (determined by Youden’s index) were calculated based on the receiver operating characteristic (ROC) curve. For the calculation of additive value of serum albumin and copeptin with the clinical scores, i.e., PSI, CURB-65 and ATS/IDSA, a binary logistic regression model was used. A two-tailed *p*-value of <0.05 was considered statistically significant.

## 4. Results

Our study included 100 patients with community-acquired pneumonia, with a mortality rate of 17%. The mean age in survivors was 54.7 ± 18.9 years and, in non-survivors, was 57.3 ± 11.5 years. The non-survivors had a higher proportion of patients with a pneumonia severity index (PSI) > 4 (*n* = 14 (77.8%)).

The non-survivors had a significantly high total WBC count (16,638.3 ± 6651.8 × 10^9^ cells/L) and absolute neutrophil count (87.2 (82.0 to 91.8) × 10^9^ cells/L)). The copeptin levels were significantly higher (33.1 ± 7.1 ng/mL) and albumin levels significantly lower (2.6 ± 0.4 g/dL) in the non-survivors compared to the survivors.

Thirty-eight patients showed clinical instability (CI) within 72 h in our study, with a male preponderance (55.3%). The mean age of CI patients was 54.7 ± 17.6 years, whereas that of non-CI patients was 55.5 ± 18.1 years. The number of ATS/IDSA minor criteria satisfied was significantly greater in the CI group compared to the non-CI group (3.0 (2.0 to 4.8) vs. 2.0 (1.0 to 4.0); *p* = 0.05). The respiratory rate was found to be significantly higher in the CI group vs. the non-CI group (27.0 (23.0 to 30.0) vs. 24.0 (20.0 to 28.0); *p* = 0.005).

The CI group had a significantly lower absolute neutrophil count (82.3 (75.2 to 88.0) vs. 86.8 (77.8 to 91.5); *p* = 0.03) and serum albumin levels (3.1 ± 0.6 vs. 3.4 ± 0.4; *p* = 0.02) and significantly high serum copeptin levels (24.0 ± 9.9 vs. 18.9 ± 7.6; *p* = 0.004). The incidence of complications was significantly higher in the CI group compared to the non-CI group (10 (26.3%) vs. 4 (6.5%); *p* = 0.013) (Table 1).

Copeptin revealed a positive correlation with PSI (ρ = 0.865 and *p* < 0.001), ATS scores (ρ = 0.295 and *p* = 0.003) and CURB-65 scores (ρ= 0.274 and *p* = 0.006). Albumin, on the other hand, correlates negatively with copeptin (r = −0.554; *p* < 0.001), PSI (ρ = −0.333; *p* < 0.001), ATS scores (ρ = −0.326; *p* < 0.001) and CURB-65 scores (ρ = −0.207; *p* = 0.038) (Table 2).

The univariate Cox proportional hazard regression analysis used to investigate the 7-day survival showed that albumin, copeptin, the presence of complications, CURB-65, ATS/IDSA and PSI were associated with significantly high hazard ratios. Regarding the multivariate analysis, however, only albumin (HR (95% CI): 0.41 (0.18–0.94, *p* = 0.034)), copeptin (HR (95% CI): 1.94 (1.03–3.67, *p* = 0.042)), the respiratory rate (HR (95% CI): 1.42 (1.08–1.88, *p* = 0.013)) and the presence of complications (HR (95% CI): 3.02 (1.02–9.25, *p* = 0.048)) were found to be strong independent predictors of 7-day mortality (Table 3).

**Figure 1:**
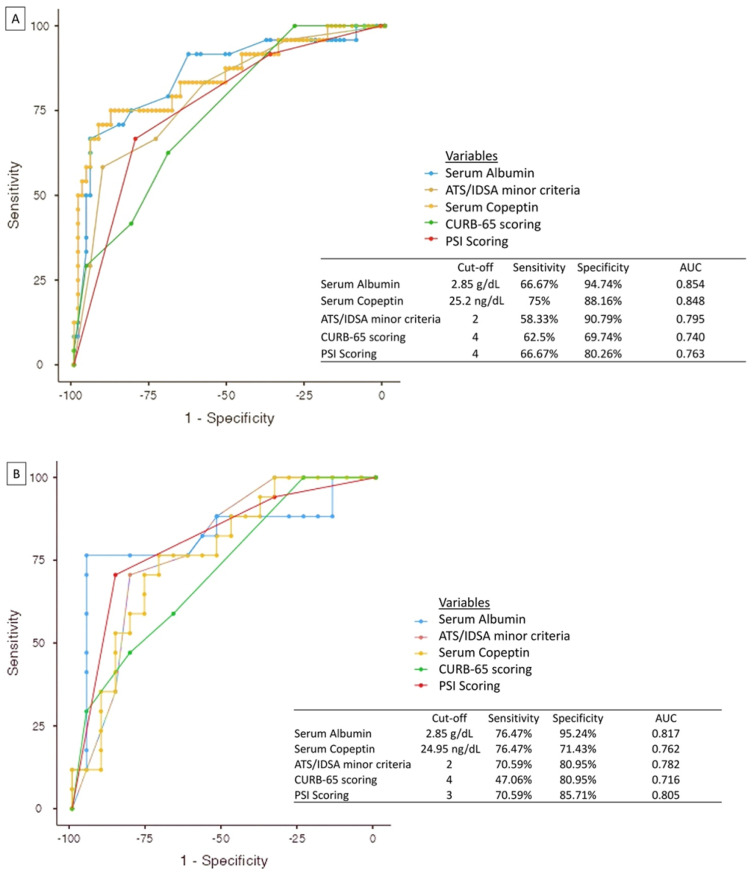
This figure depicts the results of ROC analysis for (**A**) ICU admission or 7−day mortality and (**B**) clinical instability within 72 h. Albumin, followed by copeptin, are shown to be better predictors than scoring systems in both cases. PSI = pneumonia severity index; ATS/IDSA = American Thoracic Society/Infectious Diseases Society of America; CURB−65 = confusion, urea nitrogen, respiratory rate, blood pressure, 65 years of age and older.

The ROC analysis was used to assess the prognostic value of copeptin, albumin, CURB-65, ATS/IDSA and PSI to predict ICU admission or death within 7 days. The cut-off value was 27.12 ng/mL for copeptin (sensitivity: 75.0% and specificity: 88.2%), 2.85 g/dL for albumin (sensitivity: 66.7% and specificity: 94.7%), 4.0 for PSI (sensitivity: 66.7% and specificity: 80.3%) and 3 for CURB-65 scoring (sensitivity = 62.5% and specificity 69.74%). Albumin was the best predictor of mortality (AUC = 0.854), followed by copeptin (AUC = 0.848) (Figure 1A). The prognostic power of the combined indicators calculated using the binary logistic regression model revealed that the combination of serum albumin + serum copeptin + ATS/IDSA had the highest AUC for the prediction of ICU admission or death within 7 days (AUC = 0.911; *p* = 0.007) (Table 4).

Furthermore, ROC analysis was also used to assess the prognostic value of albumin, copeptin and the scoring systems in predicting clinical instability after 72 h of admission. The cut-off for albumin remained the same at 2.85 g/dL (sensitivity: 76.5%; specificity: 95.2%), but the cut-off points for copeptin, PSI and CURB-65 scores were found to be 24.9 ng/dL (sensitivity: 76.5%; specificity: 71.4%), 3.0 (sensitivity: 70.6%; specificity: 85.8%) and 4.0 (sensitivity: 47.1%; specificity: 80.9%), respectively (Figure 1B). The prognostic power of the combined indicators (serum albumin + serum copeptin + ATS/IDSA) to predict clinical instability after 72 h of admission was the highest (AUC = 0.905; *p* = 0.019) (Table 4).

**Figure 2:**
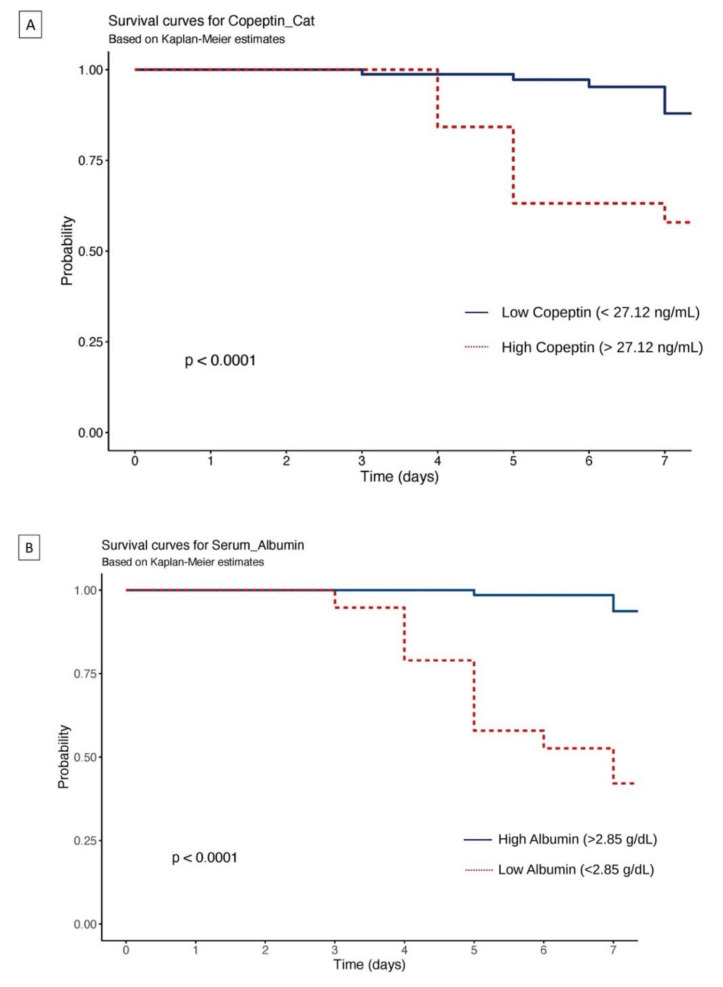
Kaplan–Meier curves showing the probability of ICU−free survival within the first 7 days after hospital admission stratified by (**A**) serum copeptin levels (**B**) serum albumin levels. *p*−value determined by log rank test.

A Kaplan–Meier analysis was performed to assess the ICU admission or death within 7 days in these patients with CAP bifurcated according to their copeptin and albumin cut-off levels identified from the ROC curves. Patients with higher copeptin (>27.12 ng/mL) saw a significantly lower (*p* < 0.001) ICU-free survival probability than those with lower copeptin levels (<27.12 ng/mL) (Figure 2A). Similar findings were observed in the Kaplan–Meier analysis for serum albumin levels. Low albumin levels (<2.85 g/dL) saw a significantly lower (*p* < 0.001) ICU-free survival probability than those with high albumin levels (>2.85 g/dL) (Figure 2B).

## 5. Discussion

We observed that increased serum albumin and copeptin levels are associated with clinical instability and early ICU admission or death in hospitalized CAP patients. Additionally, we found that the addition of these biomarkers greatly improves the prognostic value of recommended clinical scores such as PSI, ATS/IDSA criteria or the CURB-65 scoring system.

Copeptin is a protein synthesized as part of the pre-provasopressin (pre-proAVP), and the proteolytic breakdown of pre-proAVP generates the signal peptide, arginine vasopressin (AVP), neurophysin II and copeptin in the hypothalamus [24]. Stressful situations, such as various illnesses, lead to the production of the adrenocorticotropic hormone (ACTH) and cortisol from AVP, along with the corticotropin-releasing hormone (CRH). Copeptin is a hemodynamic stress and volume-dependent biomarker and elevated levels may reflect progressive sepsis or a decompensating heart or kidneys [25]. In infections of the lower respiratory tract, endotoxins and acute-phase cytokines stimulate the secretion of AVP, along with which, copeptin is synthesized in equimolar amounts. Thus, copeptin levels are significantly higher in those with an infection when compared to healthy individuals [26].

In our study, the optimal copeptin cut-off values used to predict ICU admission or death within 7 days was 27.12 ng/mL and 24.9 ng/mL for persistent clinical instability after 72 h of admission. Kolditz observed an optimal cut-off of 35 ng/dL (AUC 0.81) for predicting ICU admission or death within 7 days and persistent clinical instability after 72 h (AUC 0.74) [27], while another large study observed an optimal cut-off of 29 ng/dL (AUC 0.84) to predict 28-day mortality [28]. A systematic review and meta-analysis for predicting 30-day mortality reported that copeptin was a good independent biomarker, with a pooled AUC of 0.71 [29]. However, despite being a good prognostic indicator, copeptin gives little-to-no information about the etiology of the underlying CAP [30].

Serum albumin is an abundant plasma protein synthesized in the liver. The pathology underlying the cause of a decrease in serum albumin levels in hospitalized patients is diverse. Stress from infection, trauma, radiation or surgery has all been associated with hypoalbuminemia. Many disease states, such as solid tumors, rheumatoid arthritis and ischemic states, are associated with hypoalbuminemia [31]. Stress causes an inflammatory response and inhibits the synthesis of serum albumin in hepatocytes. Chemokines increase vascular permeability and cause the extravascular spillage of albumin, which causes hypoalbuminemia [32].

We observed that serum albumin levels lower than 2.85 mg/dL were independently associated with poor outcomes and had prognostic value for predicting ICU admission or death within 7 days (AUC = 0.854) and persistent clinical instability after 72 h (AUC = 0.817), while another large cohort study observed a cut-off value of 2.9 g/dL (AUC of 0.74) was predictive of 30-day mortality [33]. Other studies observed different thresholds, ranging from 3.0 g/dL to 3.5g/dL [34,35]. Even though the exact rationale behind the protective effect of higher serum albumin remains unknown, there are some data to suggest its role in regulating osmotic pressure in the circulatory system, hormone transport, acid–base balance and anti-apoptotic effects [32,36].

The established scoring systems, PSI and CURB-65 scores, perform well for mortality prediction and are effective in the identification of low-risk patients for outpatient management [10,11]. However, that does not translate into an accurate prediction of high-risk patients who are rapidly deteriorating or non-responders, whose early detection is crucial. In our study, we observed that CURB-65, PSI and ATS/IDSA minor criteria performed poorly compared to both serum albumin and copeptin in predicting seven-day mortality/ICU admission and clinical instability after 72 h. Serum albumin had slightly better prognostic power compared to copeptin. The ATS/IDSA minor criteria showed the highest predictive power among the scoring systems. However, they miss a relevant subset of patients who deteriorate during the course of the disease [10,14,20,36]. Thus, the impact of the addition of biomarkers to these criteria is worth exploring.

In recent years, there has been substantial research into the use of a combination of prognostic markers and scoring systems to better assess disease outcomes of CAP. When compared to the scoring systems alone, a combination of serum albumin, CRP and respiratory rate with PSI and CURB-65 significantly improved the prediction of 30-day mortality in CAP [29,37]. A study assessing clinical instability after 72 h and ICU admission or mortality at day 7 after hospital admission observed that adding serum copeptin to ATS/IDSA minor criteria improved the AUC from 0.81 to 0.85, which was similar to our study (AUC: 0.79 to 0.86). We additionally found that including both serum albumin and copeptin in ATS/IDSA minor criteria further improved the predictive power (AUC: 0.79 to 0.91).

The causes for the persistence of clinical instability, deterioration or death in CAP are multifaceted. It is a cumulative end result of various factors, such as inflammation, sepsis, shock or organ dysfunction. It can be further exacerbated by an underlying chronic disease and stress response. It has been indicated in studies that, in a setting of treatment failure, cardiac complications occur frequently in CAP [20,38]. Copeptin and albumin are potent biomarkers for predicting mid- and long-term (28 and 180 days) mortality in CAP and improving the prognostic properties of clinical scores [28,29]. Even though predictors of 28-day mortality or higher provide useful information about low risk patients suitable for earlier discharge from hospital, they fail to identify patients with early clinical deterioration or persistent clinical instability. The results of our study demonstrate that both serum albumin and copeptin predicts short-term high-risk patients in CAP.

A brief overview of the studies evaluating albumin, copeptin or both are summarized in Table 5. Very few studies have evaluated albumin and copeptin for early mortality/ICU admission and even fewer have evaluated clinical instability as an outcome. Albumin and copeptin have both been consistently observed to be a good biomarker for several outcomes of interest in CAP.

The main strengths of our study are its prospective design coupled with a well-defined patient cohort and clear study end points. This is one of the few studies that has evaluated clinical instability in patients with CAP. Our study has a few limitations. It was a single-center study with a relatively small sample size that may not be generalizable; a validation of such results needs to be carried out in large cohorts across different centers. The population is slightly skewed towards males and older people. Finally, we did not conduct serial biomarker estimation, which means we are unable to draw inferences on temporal changes in the said markers concerning hospitalization outcomes.

## 6. Conclusions

Serum albumin and copeptin can predict early mortality/ICU admission and clinical instability in hospitalized CAP patients and increase the prognostic power of traditional clinical scoring systems. These biomarkers can recognize high-risk CAP patients that are most likely to benefit from close monitoring and aggressive management.

### Clinical Implications/Future Directions

We observed that a combination of serum albumin, copeptin and ATS/IDSA criteria had the highest sensitivity and specificity in identifying subjects with risk for clinical instability, ICU admission and mortality in patients with community-acquired pneumonia. Both serum albumin and copeptin added value to the currently used clinical scores (CURB-65, PSI and ATS/IDSA criteria). Future studies need to additionally evaluate different thresholds of serum albumin and copeptin and add weighted scores to the existing scores to improve their accuracy. Serum albumin is routinely measured and more economical than serum copeptin and has the potential to be used globally, including LMIC countries with low resources. In addition, future studies need to evaluate the benefit of the supplementation of albumin on clinical stability and mortality in patients with community-acquired pneumonia and hypoalbunemia.

## Figures and Tables

**Table 1:** Baseline characteristic of study samples.

		ICU Admission/Death within 7 days			Clinical Instability within 72 h of Admission		
Outcome	Measure	Yes	No	*p*-Value	Yes	No	*p*-Value
		(*n* = 17)	(*n* = 83)		(*n* = 38)	(*n* = 62)	
Age	Mean (SD)	57.3 (11.5)	54.7 (18.9)	0.584 *	54.7 (17.6)	55.5 (18.1)	0.838 *
Sex	Male	11 (64.7%)	53 (63.9%)	0.947 ^	21 (55.3)	43 (69.4)	0.226 ^
	Female	6 (35.3%)	30 (36.1%)		17 (44.7)	19 (30.6)	
Total number of days of admission	Median (IQR)	6.5 (5.0 to 9.8)	7.0 (5.0 to 9.0)	0.917 #	7.0 (5.0 to 9.5)	6.0 (5.0 to 8.0)	0.045 #
Basal body temperature	Median (IQR)	97.8 (97.6 to 99.1)	98.7 (97.5 to 99.5)	0.381 #	98.6 (97.6 to 99.5)	98.6 (97.3 to 99.0)	0.398 #
Smokers	N (%)	7 (41.2%)	29 (34.9%)	0.626 #	16 (42.1)	20 (32.3)	0.435 #
PSI Score	Median (IQR)	5.0 (4.0 to 5.0)	4.0 (3.0 to 4.0)	<0.001 #	4.0 (4.0 to 5.0)	4.0 (3.0 to 4.0)	<0.01 #
ATS/IDSA Minor criteria	Median (IQR)	5.0 (3.0 to 6.0)	2.0 (1.0 to 4.0)	0.004 #	3.0 (2.0 to 4.8)	2.0 (1.0 to 4.0)	0.05 #
CURB-65 score	Median (IQR)	2.0 (2.0 to 3.0)	2.0 (1.0 to 2.0)	<0.001 #	2.0 (2.0 to 2.0)	2.0 (1.0 to 2.0)	0.01 #
Respiratory rate (per min)	Median (IQR)	29.0 (27.0 to 34.0)	25.0 (20.0 to 28.0)	<0.001 #	27.0 (23.0 to 30.0)	24.0 (20.0 to 28.0)	0.005 #
Comorbidities							
COPD	N (%)	6 (35.3%)	23 (27.7%)	0.53 ^	14 (36.8)	15 (24.2)	0.26 ^
Asthma	N (%)	1 (5.9%)	8 (9.6%)	0.622 ^	2 (5.3)	7 (11.3)	0.508 ^
Diabetes mellitus	N (%)	5 (29.4%)	16 (19.3%)	0.35 ^	11 (28.9)	21 (21.0)	0.202 ^
Obesity	N (%)	1 (5.9%)	4 (4.8%)	0.855 ^	1 (2.6)	4 (6.5)	0.705 ^
Hypertension	N (%)	4 (23.5%)	17 (20.5%)	0.779 ^	7 (18.4)	14 (22.6)	0.808 ^
Haematological Investigations							
Haemoglobin (g/dL)	Mean (SD)	13.3 (1.8)	12.6 (1.9)	0.172 *	13.0 (2.2)	12.6 (1.8)	0.277 *
Monocytes ×10^9^ cells/L	Mean (SD)	5.8 (2.7)	5.3 (2.3)	0.507 *	5.4 (2.6)	5.4 (2.3)	0.898 *
TLC ×10^9^ cells/L	Mean (SD)	16638.3 (6651.8)	11315.9 (5137.7)	<0.001 *	13452.1 (6400.9)	11551.8 (5290.5)	0.111 *
ANC ×10^9^ cells/L	Median (IQR)	87.2 (82.0 to 91.8)	83.7 (77.0 to 89.0)	0.111 #	82.3 (75.2 to 88.0)	86.8 (77.8 to 91.5)	0.03 #
ALC ×10^9^ cells/L	Median (IQR)	8.6 (6.0 to 13.0)	9.9 (6.7 to 16.8)	0.476 #	11.0 (7.6 to 17.6)	9.0 (6.0 to 15.2)	0.092 #
Platelets (lakh cells/cu.mm)	Median (IQR)	2.7 (1.9 to 3.1)	2.7 (2.3 to 3.3)	0.481 #	2.7 (2.2 to 3.3)	2.7 (2.1 to 3.5)	0.699 #
NLR	Median (IQR)	10.1 (6.4 to 15.3)	8.5 (4.6 to 13.4)	0.419 #	7.5 (4.6 to 11.7)	9.8 (5.1 to 15.2)	0.079 #
PLR	Median (IQR)	0.2 (0.2 to 0.3)	0.3 (0.2 to 0.4)	0.6 #	0.2 (0.2 to 0.4)	0.3 (0.2 to 0.5)	0.076 #
CRP (mg/L)	Mean (SD)	29.2 (13.6)	23.1 (13.0)	0.079 *	24.8 (12.5)	23.9 (13.7)	0.75 *
Serum Albumin (g/dL)	Mean (SD)	2.6 (0.4)	3.4 (0.4)	<0.001 *	3.1 (0.6)	3.4 (0.4)	0.002 *
Serum Copeptin (ng/mL)	Mean (SD)	33.1 (7.1)	18.1 (6.7)	<0.001 *	24.0 (9.9)	18.9 (7.6)	0.004 *
Complications **	Yes	8 (44.4)	4 (4.9)	<0.001 ^	10 (26.3)	4 (6.5)	0.013 ^
	No	10 (55.6)	78 (95.1)		28 (73.7)	58 (93.5)	

‘*n*’ is the number of non-missing values. # Wilcoxon ^ Pearson chi sq. * Student’s *t*. PSI = pneumonia severity index; ATS/IDSA = American Thoracic Society/Infectious Diseases Society of America; CURB-65 = confusion, urea nitrogen, respiratory rate, blood pressure, 65 years of age and older; COPD = chronic obstructive pulmonary disease; TLC = total leukocyte count; ANC = absolute neutrophil count; ALC = absolute lymphocyte count; NLR = neutrophil-to-lymphocyte ratio; PLR = platelet-to-lymphocyte ratio; CRP = C-reactive protein. ** Complications include pleural effusion, multiple organ dysfunction syndrome (MODS) and septic shock.

**Table 2:** Correlation matrix.

		Albumin	Copeptin	PSI	ATS	CURB-65
Albumin	Pearson’s r	-				
	*p*-value	-				
	95% CI Upper	-				
	95% CI Lower	-				
	Spearman’s rho	-				
	*p*-value	-				
Copeptin	Pearson’s r	−0.554 ***	-			
	*p*-value	<0.001	-			
	95% CI Upper	−0.401	-			
	95% CI Lower	−0.677	-			
PSI	Spearman’s rho	−0.333 ***	0.865 ***	-		
	*p*-value	<0.001	<0.001	-		
	95% CI Upper	−0.137	0.88	-		
	95% CI Lower	−0.49	0.751	-		
ATS/IDSA	Spearman’s rho	−0.326 ***	0.295 **	0.22 *	-	
	*p*-value	< .001	0.003	0.028	-	
	95% CI Upper	−0.195	0.457	0.39	-	
	95% CI Lower	−0.534	0.095	0.013	-	
CURB-65	Spearman’s rho	−0.207 *	0.274 **	0.209 *	0.822 ***	0.539 ***
	*p*-value	0.038	0.006	0.037	<0.001	<0.001
	95% CI Upper	−0.051	0.432	0.366	0.861	0.552
	95% CI Lower	−0.421	0.065	−0.014	0.716	0.22

* *p* < 0.05 ** *p* <0.01 *** *p* < 0.001; PSI = pneumonia severity index; ATS/IDSA = American Thoracic Society/Infectious Diseases Society of America; CURB-65 = confusion, urea nitrogen, respiratory rate, blood pressure, 65 years of age and older.

**Table 3:** Cox regression analysis of risk factors associated with 7-day mortality/ ICU admission.

Dependent	HR (Univariable)	HR (Multivariable)
PSI	2.06 (1.06–4.03, *p* = 0.034)	1.31 (0.61–2.78, *p* = 0.487)
CURB-65	11.31 (1.25–102.27, *p* = 0.031)	8.92 (0.74–107.35, *p* = 0.085)
ATS/IDSA	7.16 (1.01–56.10, *p* = 0.041)	1.35 (0.06–30.93, *p* = 0.849)
Respiratory rate	1.12 (1.05–1.20, *p* < 0.001)	1.42 (1.08-1.88, *p* = 0.013)
TLC	1.00 (1.00–1.00, *p* = 0.113)	1.00 (1.00–1.00, *p* = 0.873)
Complications *	5.35 (2.26–12.68, *p* < 0.001)	3.02 (1.02–9.25, *p* = 0.048)
Albumin	0.28 (0.15–0.54, *p* < 0.001)	0.41 (0.18–0.94, *p* = 0.034)
Copeptin	1.09 (1.04–1.15, *p* ≤0.001)	1.94 (1.03–3.67, *p* = 0.042)

* Complications include pleural effusion, multiple organ dysfunction syndrome (MODS) and septic shock; PSI = pneumonia severity index; ATS/IDSA = American Thoracic Society/Infectious Diseases Society of America; CURB-65 = confusion, urea nitrogen, respiratory rate, blood pressure, 65 years of age and older; TLC: total leucocyte count.

**Table 4:** Receiver operating characteristic curves for the combined use of the scoring systems with albumin and copeptin for predicting clinical instability after 72 h and ICU admission or mortality at day 7 after hospital admission.

Clinical Instability				
Predictor	SENS	SPE	AUC	*p*-Value
Serum Albumin + PSI	0.857	0.706	0.885	0.021 *
Serum Albumin + ATS/IDSA	0.857	0.765	0.894	0.012 *
Serum Albumin + CURB-65	0.81	0.765	0.838	0.15
Serum Copeptin + PSI	0.81	0.706	0.84	0.026 *
Serum Copeptin + ATS/IDSA	0.857	0.706	0.863	0.007 *
Serum Copeptin + CURB-65	0.81	0.647	0.849	0.026 *
Serum Albumin + Serum Copeptin + PSI	0.857	0.765	0.894	0.043 *
Serum Albumin + Serum Copeptin + ATS/IDSA	0.857	0.785	0.905	0.019 *
Serum Albumin + Serum Copeptin + CURB-65	0.857	0.706	0.885	0.096 *
**Mortality/ICU admission in 7 days**				
**Predictor**	**SENS**	**SPE**	**AUC**	***p*** **-value**
Serum Albumin + PSI	0.947	0.625	0.89	0.010 *
Serum Albumin + ATS/IDSA	0.934	0.583	0.885	0.004 *
Serum Albumin + CURB-65	0.947	0.542	0.897	0.061 *
Serum Copeptin + PSI	0.974	0.542	0.852	0.351
Serum Copeptin + ATS/IDSA	0.961	0.708	0.898	0.001 *
Serum Copeptin + CURB-65	0.974	0.583	0.864	0.075 *
Serum Albumin + Serum Copeptin + PSI	0.947	0.667	0.888	0.942
Serum Albumin + Serum Copeptin + ATS/IDSA	0.947	0.75	0.911	0.007 *
Serum Albumin + Serum Copeptin + CURB-65	0.947	0.667	0.895	0.013 *

* *p* < 0.05; SENS = sensitivity; SPE = specificity; AUC = area under the curve. PSI = pneumonia severity index; ATS/IDSA = American Thoracic Society/Infectious Diseases Society of America; CURB-65 = confusion, urea nitrogen, respiratory rate, blood pressure, 65 years of age and older.

**Table 5:** Various studies evaluating albumin, copeptin or both for various outcomes of CAP.

Author	Sample Size	Marker	Outcome	Result
Present study, 2021, India	100	Albumin, copeptin	Combined ICU admission or mortality at day 7 and clinical instability after 72 h.	Albumin was the best predictor of mortality (AUC = 0.854), followed by copeptin (AUC = 0.848). The combination of serum albumin + serum copeptin + ATS/IDSA had the highest AUC for prediction of ICU admission or death within 7 days (AUC = 0.911) and for prediction of clinical instability after 72 h of admission (AUC = 0.905).
Zhao L et al. [37], 2021, China	366	Albumin	30-day mortality	PSI and CURB-65 were found to be better independent predictors compared to albumin (AUC: 0.79 and 0.78 vs. 0.76)
Avci S et al. [39], 2020, Turkey	206	Albumin, c-reactive protein/albumin ratio (CAR), CRP, NLR, PLR, procalcitonin, A-a o2 gradient, A-a o2 difference	Prediction of 30-day mortality	Albumin (AUC: 0.80) was found to be a better predictor of 30-day mortality than all blood parameters and CURB-65 scores. PSI score was found to be better than albumin (AUC: 0.86)
Celikhisar H et al. [34], 2020, Turkey	86	Lactate, procalcitonin, blood glucose, serum albumin	Risk factors for ICU mortality	Lactate, procalcitonin, albumin (OR: 3.34) and blood glucose were found to be significant independent risk factors
Shi T et al. [35], 2020, China	113	Albumin, ALT, AST, hemoglobin	Risk factors for mortality	<90% oxygen saturation and albumin < 35 g/L were found to be significant risk factors for mortality (OR: 8.77 and 4.73, respectively)
He Y et al. [40], 2019, China	175	WBC count, CRP, procalcitonin, hemoglobin, platelet count, albumin, BUN, creatinine, uric acid, AST and ALT	Risk factors for severe pneumonia	On admission, albumin was significantly lower in SCAP patients compared to non-severe patients
Adnan M et al. [41], 2018, Pakistan	134	Albumin, B/A ratio, BUN	Prediction of ICU admission	Albumin was found to be the better predictor for ICU admission (AUC: 0.718) compared to B/A ratio, BUN or CURB-65
Miyazaki H et al. [42], 2018, Japan	534	Albumin, PCT	30-day mortality	Lowest albumin within 1 week of admission was found to be the best predictor (AUC:0.85), followed by albumin at admission (0.81)
Curbelo J et al. [43], 2017, Spain	154	Copeptin, proadrenomedullin, lymphocyte%, neutrophil%, NLR, procalcitonin	Prediction of 30-day and 90-day mortality	On admission, pro-ADM followed by copeptin were found to be the best predictors of both 30-day (AUC: 0.89 and 0.84, respectively) and 90-day (AUC: 0.84 and 0.79, respectively) mortality
Holter JC et al. [44], 2016, Norway	259	CRP, creatinine, albumin	Prediction of risk of long-term mortality	It was found that every 5 g/L decrease in albumin levels increased the risk of death by 25%.
Alcoba G et. [45], 2015, Switzerland	88	Copeptin, proadrenomedullin, CRP	Prediction of complicated pneumonia	Proadrenomedullin and CRP performed much better than copeptin in predicting complicated pneumonia (AUC: 0.85 each vs. 0.59)
Kruger S et al. [28], 2013, Germany	1740	CRP, procalcitonin, MR-proadrenomedullin, WBC count and copeptin	28-day mortality and 180-day mortality	Copeptin was found to have superior diagnostic accuracy to MR-proANP, CRP, CRB-65 and WBC count (AUC: 0.84) in prediction of 28-day mortality. Similar results were found for 180-day mortality (AUC: 0.78), except for MR-proANP (AUC: 0.81)
Kolditz M et al. [27], 2012, Germany	51	Copeptin, C-reactive protein, MR-proadrenomedullin and pro-calcitonin	Combined ICU admission or mortality at day 7 and clinical instability after 72 h.	The diagnostic accuracy of copeptin for both ICU admission and mortality, as well as clinical instability (AUC: 0.81 and 0.74, respectively). Addition of copeptin to ATS/IDSA minor criteria resulted in significant improvement in prediction of 7-day mortality (AUC: 0.85) and prediction of clinical instability (combined AUC: 0.81).
Suter-Widmer I et al. [46], 2012, Switzerland	875	Albumin, CRP, procalcitonin	Prediction of length of hospital stay	Albumin was associated with increased duration of hospital stay (HR: 0.77)
Lee JH et al. [47], 2011, South Korea	424	Albumin, CRP	28-day mortality, ICU admission or vasopressor use or mechanical ventilation	Albumin was found to be the best predictor of 28-day mortality (AUC: 0.66). When added with PSI and CRP, the predictive capability significantly increased (AUC: 0.76)
Schuetz P et al. [48], 2010, Switzerland	925	Procalcitonin, pro-ADM, pro-ANP, pro-ET1, copeptin	Prediction of severe outcome	Copeptin (AUC: 0.70) was superior to PSI and curb-65 alone but inferior to pro-ET1 and pro-ADM (AUC: 0.72, respectively)
Muller B et al. [26], 2007, Germany	545	Copeptin, CRP, leucocyte count, procalcitonin	6-week mortality	Copeptin was found to be the better predictor (AUC: 0.75)

## Data Availability

All data generated or analyzed during this study are included in this published article and are available from the corresponding author upon reasonable request.
